# Differential Inflammatory MicroRNA and Cytokine Expression in Pulmonary Sarcoidosis

**DOI:** 10.1007/s00005-014-0315-9

**Published:** 2014-11-01

**Authors:** Agnieszka Jazwa, Lukasz Kasper, Maciej Bak, Mateusz Sobczak, Krzysztof Szade, Alicja Jozkowicz, Krzysztof Sladek, Jozef Dulak

**Affiliations:** 1 Department of Medical Biotechnology, Faculty of Biochemistry, Biophysics and Biotechnology, Jagiellonian University, Gronostajowa 7, 30-387 Kraków, Poland; 2 Department of Pulmonology, II Department of Medicine, Jagiellonian University Medical College, Skawinska 8, 31-066 Kraków, Poland

**Keywords:** Sarcoidosis, miRNA, miR-34a, IFN-γ, Vascular endothelial growth factor, Inflammation

## Abstract

Sarcoidosis is a granulomatous disease of unknown etiology. The disease has an important inflammatory and immune component; however, its immunopathogenesis is not completely understood. Recently, the role of microRNAs (miRNAs), the small non-coding RNAs, has attracted attention as both being involved in pathogenesis and serving as disease markers. Accordingly, changes in the expression of some miRNAs have been also associated with different autoimmune pathologies. However, not much is known about the role of miRNAs in sarcoidosis. Therefore, the aim of this study was to compare the level of expression of selected miRNAs in healthy individuals and patients with sarcoidosis. We detected significantly increased level of miR-34a in peripheral blood mononuclear cells isolated from sarcoidosis patients. Moreover, significantly up-regulated levels of interferon (IFN)-γ, IFN-γ inducible protein (IP-10) and vascular endothelial growth factor were detected in sera of patients when compared to healthy subjects. Our results add to a known inflammatory component in sarcoidosis. Changes in the levels of miR-34a may suggest its involvement in the pathology of this disease.

## Introduction

Sarcoidosis is a granulomatous disease of unknown etiology that affects people all around the world (Hunninghake et al. 1999). Although changes might be seen in almost all organs, in approximately 90 % of sarcoidosis patients non-necrotizing granulomas are found in the lungs. Over the last 10 years, there has been a substantial progress on the etiopathogenesis, diagnosis and treatment of the disease (Baughman et al. 2011; Rossman and Kreider 2007). It is well accepted that sarcoidosis has an important inflammatory component and can be defined as enhanced, immunologic hypersensitivity to pathogenic tissue antigens with CD4^+^ T-cell infiltration (Moller and Chen 2002). Sarcoidosis demonstrates well-documented, polarized Th1 cytokine, chemokine and chemokine receptor expression, with oligoclonal expansion of T-cell receptor αβ^+^ T cells consistent with conventional antigen stimulation and activation of macrophages in non-fibrotic disease. Antigens associated with sarcoidosis are still unknown, but there are data suggesting that autoantigens such as vimentin and ATP-synthase (Wahlstrom et al. 2007) may be the targets for expanded T-cell activation in pathology of the disease. There are also data suggesting that some bacteria such as *Mycobacterium tuberculosis* and *Propionibacterium* spp. (Eishi et al. 2002; Song et al. 2005), as well as some non-infectious antigens, such as dusts, gases, organic particles, industrial pollution and beryllium (Sawyer and Maier 2011), may also be responsible for developing the disease in susceptible people. It is suggested that certain HLA alleles are responsible for susceptibility to disease development, some are involved in chronic and recurrent types of disease and some contribute to acute sarcoidosis and fast disease resolution (Rossman et al. 2003).

MicroRNAs (miRNAs) have recently emerged as a new class of modulators of gene expression. They are small (approximately 22 nucleotides in length), non-coding RNAs that regulate gene expression, mainly at the post-transcriptional level, via degradation or translational inhibition of their target mRNAs (Ciesla et al. 2011). Approximately 500–1,000 miRNAs are expressed in human cells (although current estimations reach even the number of 3,000), and their expression signatures vary depending on the tissue and cell types examined. These molecules have important functions in the immune system, such as regulation of hematopoietic development, cancer and immune homeostasis. Changes of expression of some miRNAs have been reported in autoimmune pathologies such as rheumatoid arthritis, systemic lupus erythematosus, and multiple sclerosis (Ha 2011; O’Connell et al. 2012). Extracellular miRNAs in serum or plasma have been also recently suggested as markers for monitoring disease process and effectiveness of therapy (Ciesla et al. 2011).

Despite intensive studies on the regulation of inflammatory and immune response by miRNAs and the acceptance of the significant immune component in pathogenesis of sarcoidosis, the studies on the role of miRNAs in this disease are scarce. Only very recently, two papers have been published. In the first study, the miRNAs regulating TGF-β pathways, previously also incriminated in pathogenesis of sarcoidosis, have been elucidated (Crouser et al. 2012). The second, more extensive analysis (Maertzdorf et al. 2012), revealed similarity in gene expression signatures of tuberculosis with that of sarcoidosis, confirming the suggested link for etiopathogenesis of this disease. Importantly, in both conditions, the up-regulation of pro-inflammatory pathways has been observed. Therefore, the aim of this study was to extend the knowledge on the role of miRNAs in sarcoidosis and to compare the level of expression of selected inflammatory miRNAs in healthy individuals and patients with this disease.

## Materials and Methods

### Subjects

Peripheral blood was collected from 12 patients with pulmonary sarcoidosis and five control, age- and gender-matched healthy subjects at the Department of Pulmonology, II Department of Medicine, Jagiellonian University Medical College in Krakow. Diagnosis of sarcoidosis was determined in compliance with international criteria (Hunninghake et al. 1999). There were eight men and four female subjects studied and the mean age of this population was 46 years: the youngest patient was 31, the oldest 67 years old (Table 1). Mean disease duration was 5 years (minimum 12 months, the longest 10 years). In the studied group, there were five patients with acute sarcoidosis and seven with chronic form of the disease (Table 1). In patients with an acute form during the 2 years of follow-up, there has been a self-remission, and they did not require treatment. All of the patients with chronic sarcoidosis required prolonged corticosteroid therapy during the course of disease. The mean time of steroid treatment was 30 months (minimum 12 months, maximum 8 years; Table 2). Subjects received routine therapy at an initial dose of 32 mg of methylprednisolone through systematic dosage reduction to complete withdrawal. One patient with the longest duration of treatment received little maintenance dose (4 mg of methylprednisolone) to prevent recurrence of the disease. None of the patients received steroid therapy at the time of blood sampling. In one case of acute sarcoidosis, we decided to start treatment just after, because of rapid radiological progression in lung tissue. In this particular case, we observed very high level of pro-inflammatory cytokines during our study. Other two patients with very high level of these cytokines might be also clinically characterized as a chronic active sarcoidosis (with relapses of the disease). Other patients had sarcoidosis disease in remission (without radiological progression and decline in lung function). All patients gave their informed consent for the use of peripheral blood for the purpose of this study. The local ethical committee of the Jagiellonian University (Krakow, Poland) approved the study.

**Table 1:** General characteristics of the study population

Number of patients	12 (8 male, 4 female)
Mean age	46 (31–67)
Mean disease duration	5 years (12 months, 10 years)
Acute sarcoidosis	5
Chronic sarcoidosis	7

**Table 2:** Clinical status of patients with sarcoidosis

	Radiological classification (no.)	Steroid treatment (no.)	Mean time of treatment	Lung function VC/DLco
Acute disease	Stage I (4)	0	0	Normal
	Stage II (1)	0	0	Normal
Chronic disease	Stage II (6)	6	2 years	
	Stage IV (1)	1	8 years	

*VC* vital capacity, *DLco* diffusion capacity of the lung for carbon monoxide

### Serum Preparation

The venous blood (5 ml) was collected to the vacuum gel tubes and allowed to stand for approximately 30 min in room temperature before being centrifuged at 3,500 rpm for 10 min. The resulting serum was aliquoted into Eppendorf tubes and stored at −80 °C until further use.

### Analysis of Serum Inflammatory Cytokines with FlexMap3D Luminex System

To determine the concentration of a panel of inflammatory cytokines (EGF, Eotaxin, FGF basic, G-CSF, GM-CSF, HGF, IFN-α, IFN-γ, IL-1RA, IL-1β, IL-2, IL-2R, IL-4, IL-5, IL-6, IL-7, IL-8, IL-10, IL-12 (p40/p70), IL-13, IL-15, IL-17, IP-10, MCP-1, MIG, MIP-1α, MIP-1β, RANTES, TNF-α, and VEGF) in the patients’ sera, FlexMap3D luminex was applied. The analysis was performed using the Human Cytokine Magnetic 30-Plex Panel (Invitrogen, Poland) according to the manufacturer’s instructions. In total, 11 serum samples from sarcoidosis patients and five serum samples from healthy donors were tested. The results were analyzed with FlexMap3D xPONENT software.

### Isolation of Peripheral Blood Mononuclear Cells

Peripheral blood mononuclear cells (PBMCs) were isolated as described previously (Bazan-Socha et al. 2012). Briefly, 20–25 ml blood was drawn into EDTA-coated tubes from the antecubital vein of 12 sarcoidosis patients and 5 healthy volunteers. PBMCs were isolated by Histopaque (Sigma-Aldrich) density gradient centrifugation (400×*g*, 30 min, 20 °C). Cells were washed twice in RPMI 1,640 medium and were counted. The whole processing time was approximately 90 min. PBMCs were stored frozen (20 % FBS, 10 % DMSO) in a liquid nitrogen until further use.

### RNA Isolation

Total RNA containing miRNA fraction was isolated from serum (200 µl) and from PBMCs by lysis in 1 ml of Qiazol Total RNA Isolation Reagent using Tissue Lyzer (Qiagen, Poland). To allow for normalization of sample-to-sample variation in the RNA isolation step from serum, synthetic *C. elegans* miRNA cel-miR-39 (synthetic RNA oligonucleotide 5′-UCACCGGGUGUAAAUCAGCUUG-3′ synthesized by Institute of Biochemistry and Biophysics, Polish Academy of Sciences) was added (25 fmol of the oligonucleotide in a 5 µl total volume) to each denatured sample (shortly after combining the serum sample with Qiazol). Then, samples were supplied with 200 µl of chloroform, the mixture was vortexed (30 s), incubated on ice (20 min) and centrifuged (12,000×*g*, 20 min, 4 °C). Then, the aqueous phase was transferred to new Eppendorf tubes, mixed with equal amount of isopropanol (in case of PBMCs) or 100 % ethanol (in case of serum), incubated overnight at −20 °C, and centrifuged (10,000×*g*, 30 min, 4 °C). Pellets were washed twice with ice-cold 70 % ethanol and centrifuged (10,000×*g*, 10 min, 4 °C). Finally, the pellets were air dried and resuspended in 10 µl of nuclease-free water. Concentration and quality of RNA was determined only in PBMCs isolates by measuring absorbance at 260 and 280 nm.

### miRNA Reverse Transcription and Quantitative PCR

cDNA template was synthesized from 500 ng of total RNA isolated from PBMCs and from the whole volume of RNA sample isolated from each serum using NCode miRNA First-Strand cDNA Synthesis Kit (Invitrogen, Poland) following the manufacturer protocol. Obtained cDNA from PBMCs samples was diluted five times in ultrapure water. Quantitative PCR (qPCR) was performed using StepOne Plus Real-Time PCR (Applied Biosystems, Poland) in a mixture containing SYBR Green PCR Master Mix (SYBR Green qPCR Kit, Sigma, Poland), 20 ng of cDNA (in case of PBMC samples) and two primers: a specific miRNA forward primer (Table 3) and a universal reverse primer for miRNAs qPCR supplied by the vendor (Invitrogen, Poland) in a total volume of 15 μl. In case of serum samples, preamplification step was performed as follows: 5-μl aliquot of undiluted cDNA was combined with 15 μl of preamplification PCR reagents [comprised, per reaction, of 0.1 μl of Taq DNA polymerase, 2 μl of enzyme buffer (10×), 0.4 μl of dNTP, 2 μl of a mixture of several forward primers (final concentration of each primer 100 nM) amplifying each specific miRNA (Table 3), 0.2 μl of universal qPCR reverse primer (Invitrogen, Poland) and 10.3 μl of water]. Preamplification PCR was carried out in the MasterCycler Personal Thermal Cycler (Eppendorf, Poland) by heating to 95 °C for 5 min, followed by 15 cycles of 95 °C for 30 s, 60 °C for 30 s and 72 °C for 30 s. The preamplification mixture was diluted (by adding 180 μl of ultrapure water to the 20 μl preamplification reaction product), following which 2 μl of diluted material was introduced into the real-time PCR. MiRNA expression in each tested serum was normalized to introduced synthetic cel-miR-39. Each miRNA from PBMCs was normalized to a constitutive small RNA U6. Melting curve analysis was done using the program supplied by Applied Biosystems (Poland). Samples with unspecific amplification product (primer dimers) were excluded from the analysis. Relative quantification of miRNA expression was calculated based on comparative *C*_T_ (threshold cycle value) method (Δ*C*_T_ = *C*_T miRNA of interest_ − *C*_T reference miRNA_). Values are shown as 2^−ΔCT^.

**Table 3:** Sequences of forward primers for amplification of miRNAs with qPCR

miRNA	Sequence
miR-15a	5′-CGCTAGCAGCACATAATGGTTTGTG-3′
miR-16	5′-CGTAGCAGCACGTAAATATTGGCG-3′
miR-34a	5′-TGGCAGTGTCTTAGCTGGTTGT-3′
miR-146a	5′-CGTGAGAACTGAATTCCATGGGTT-3′
miR-150	5′-TCTCCCAACCCTTGTACCAGTG-3′
miR-155	5′-TTAATGCTAATCGTGATAGGGGTA-3′
miR-192	5′-CTGACCTATGAATTGACAGCC-3′
miR-326	5′-CCTCTGGGCCCTTCCTCCAG-3′
miR-378	5′-ACTGGACTTGGAGTCAGAAGG-3′
cel-miR-39	5′-TCACCGGGTGTAAATCAGCTTG-3′

### Data Analysis

Results are expressed as median with or without range. Non-parametric Mann–Whitney test was used to evaluate the statistical significance between investigated groups. *p* < 0.05 was considered as statistically significant.

## Results

### Serum Cytokines

**Fig. 1:**
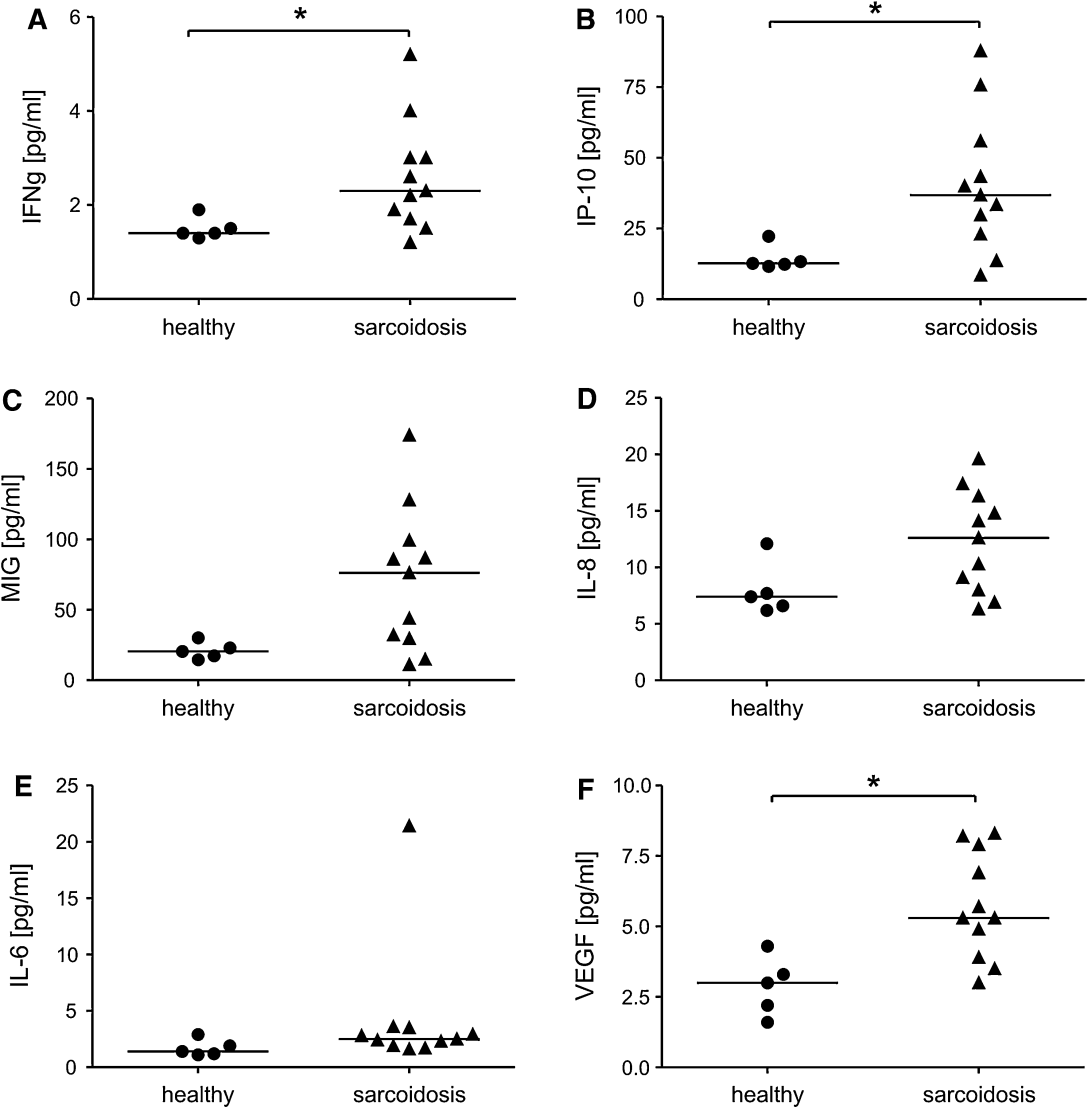
Serum cytokine and chemokine levels in diseased and healthy individuals. Luminex assay. **a** IFN-γ, **b** IFN-γ inducible protein (IP-10); **c** monokine induced by IFN-γ (MIG); **d** IL-8; **e** IL-6; **f** VEGF. *Significant difference between indicated groups (median) at *p* < 0.05 (Mann–Whitney test)

We measured a total of 30 serum analytes in the sera of sarcoidosis patients and healthy controls to define the general immune activity in each patient and differences between the diseased and healthy study groups. Sarcoidosis is known as a Th1-mediated disease with IFN-γ production in the lungs (Moller et al. 1996). Sera from sarcoidosis patients showed increase in this pro-inflammatory cytokine (Fig. 1a, *p* < 0.05) as well as IFN-γ-related CXC chemokine IFN-γ inducible protein (IP-10, Fig. 1b, *p* < 0.05). Additionally, we observed a trend towards higher level of monokine induced by IFN-γ (MIG, Fig. 1c, *p* = 0.05). Both of these cytokines, IP-10 and MIG, were described as pro-inflammatory and anti-angiogenic agents. Additionally, we have observed a trend towards increased levels of IL-8 (Fig. 1d, *p* = 0.05) and IL-6 (Fig. 1e, *p* = 0.05) in patients with pulmonary sarcoidosis. Finally, sera from sarcoidosis patients showed significant up-regulation of vascular endothelial growth factor (VEGF, Fig. 1f, *p* < 0.05), a major regulator of angiogenesis, proposed to be useful prognostic marker of sarcoidosis (Sekiya et al. 2003).

### Peripheral Blood miRNAs

Several small non-coding RNAs were shown to play an important role in the mechanisms of both innate and adaptive immunity (O’Connell et al. 2012). Therefore, in the present study, we were interested in checking the expression of some of the inflammation-associated miRNAs in PBMCs and serum of patients with pulmonary sarcoidosis. None of the eight analyzed serum miRNAs was relatively invariant (with an SD less than 1) across all experimental samples (Table 4, range). Therefore, as a reference we used the introduced synthetic miRNA corresponding to *Caenorhabditis elegans* miRNA (*cel*-*miR*-*39*), chosen because of the absence of homologous sequence in humans. This synthetic miRNA was spiked to human serum after addition of a denaturing solution that inhibits RNase activity. Preamplification step was introduced to increase the sensitivity of detection of sera miRNAs in qPCR. Among eight analyzed serum miRNAs, we did not find any statistically significant differences (Table 4).

**Table 4:** Median 2^−ΔCT^ values of miRNAs analyzed in serum of two populations

miRNA	Healthy median/range	Sarcoidosis median/range	*p* value
miR-15a	0.17/0.04–0.27	0.1/0.05–0.3	NS
miR-16	7.04/3.39–18.14	8.23/1.55–38.73	NS
miR-34a	0.49/0.29–0.97	0.63/0.1–1.99	NS
miR-146a	2.26/0.72–7.64	2.83/0.18–6.78	NS
miR-150	5.92/1.83–26.01	2.87/0.73–7.27	NS
miR-155	0.0016/0.0004–0.0058	0.0075/0.0003–0.0316	NS
miR-192	0.81/0.45–1.29	1.11/0.09–3.05	NS
miR-378	1.76/1.54–2.47	3.06479/0.27–14.41	NS

*NS* not significant

**Fig. 2:**
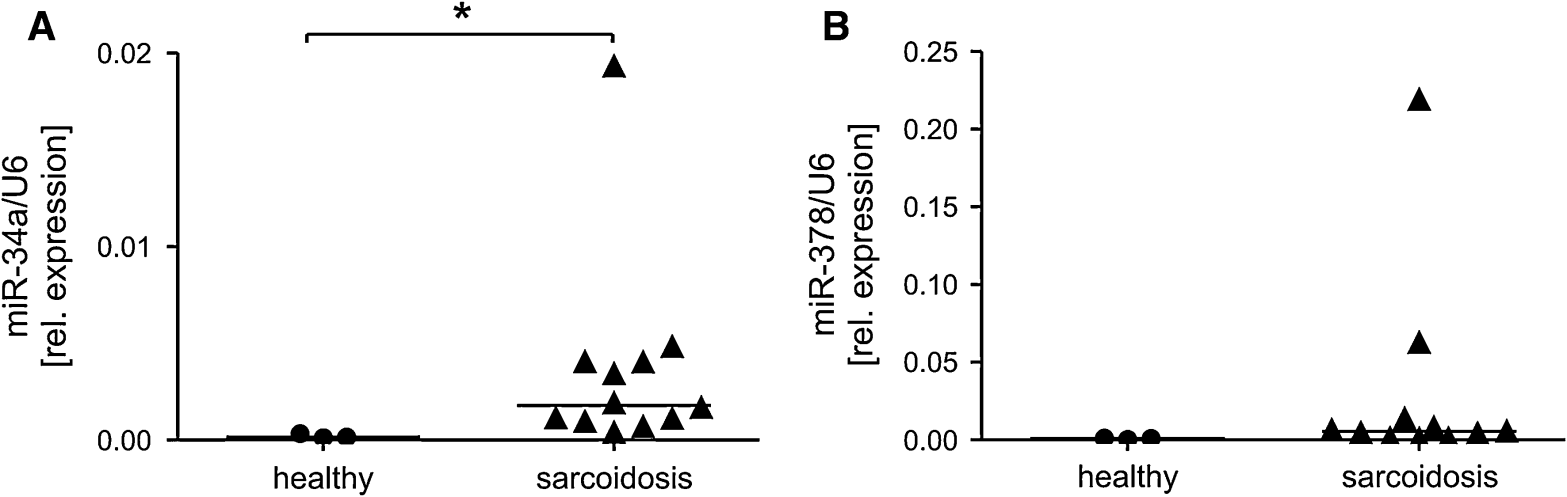
Relative expression of selected miRNAs in PBMC. **a** miR-34a, **b** miR-378. The level of both miRNAs was normalized to small nuclear U6. *Significant difference between indicated groups (median) at *p* < 0.05 (Mann–Whitney test)

The very similar miRNA profile was analyzed in PBMCs of healthy and diseased individuals (Table 5). Significantly higher levels of miR-34a were detected in PBMCs isolated from sarcoidosis patients when compared to healthy controls (Table 5; Fig. 2a). Interestingly, the level of miR-378 tended to be higher (*p* = 0.06) in PBMCs of sarcoidosis patients (Table 5; Fig. 2b).

**Table 5:** Median 2^−ΔCT^ values of miRNAs analyzed in PBMCs of two populations

miRNA	Healthy median/range	Sarcoidosis median/range	*p* value
miR-15a	0.248/0.007–0.838	0.194/0.082–1.925	NS
miR-16	0.006/0.001–1.572	0.029/0.007–1.967	NS
miR-34a	0.00015/0.0001–0.00035	0.0018/0.0004–0.0193	<0.05
miR-146a	0.0922/0.00005–0.3767	0.0276/0.00004–0.2723	NS
miR-150	0.117/0.023–0.314	0.151/0.008–1.268	NS
miR-155	0.0627/0.00004–0.2382	0.0176/0.0025–0.376	NS
miR-326	0.78/0.02–0.99	0.32/0.05–1.535	NS
miR-378	0.0009/0.0003–0.0011	0.0056/0.0006–0.219	NS

*NS* not significant

## Discussion

Although diagnostic strategies and management of sarcoidosis have improved, determining the causes and population at risk for the disease would not only enhance diagnosis and treatment but, possibly may aid in prevention. Therefore, in the present study, we tried to determine whether serum proteins and miRNAs might be useful in distinguishing healthy individuals from patients with sarcoidosis.

Analysis of median levels of inflammatory cytokines confirmed previously published studies showing up-regulation of IFN-γ, IP-10 (Beirne et al. 2009; Kopinski et al. 2007; Qazi et al. 2010) and VEGF (Sekiya et al. 2003) in sarcoidosis. Moreover, we observed a trend towards higher levels of MIG, IL-8, and IL-6, which were previously shown to be increased in sarcoidosis (Beirne et al. 2009). Also, other proteins such as IL-12 (Beirne et al. 2009; Shigehara et al. 2003), MCP-1 and MIP-1 (Beirne et al. 2009; Hashimoto et al. 1998) previously reported as up-regulated in sarcoidosis, did not differ significantly between our studied populations. The lack of statistically significant changes was observed also when we divided sarcoidosis patients into two groups: with acute and chronic form of the disease, and compared each of them with healthy individuals. In both groups, we found patients who had extremely or relatively high and low levels of inflammatory cytokines. The reasons for this discrepancy between our and other (Hashimoto et al. 1998; Beirne et al. 2009; Shigehara et al. 2003) findings could include assay sensitivity and/or choice of patient population and their number analyzed. However, lack of specificity and sensitivity of many of the current blood biomarkers impedes the definitive disease diagnosis. Such difficulties may also occur when it comes to differentiation of sarcoidosis from other infectious and non-infectious granulomatous diseases.

Extracellular miRNAs present in blood show remarkable stability and resistance to degradation (Mitchell et al. 2008). Initially, it was proposed that this is because miRNAs are enclosed in microvesicles (e.g., microparticles, exosomes), forming a physical barrier against RNase activity. Later studies revealed that miRNAs can interact with proteins such as Ago2 and LDL- or HDL-lipoproteins and in this way can also be protected against RNase-mediated degradation (reviewed in Zampetaki and Mayr 2012). Indeed, miRNAs are suggested to have a potential as biomarkers for various pathophysiological conditions (Ciesla et al. 2011) but, so far, very little is known about their role in sarcoidosis (Crouser et al. 2012; Maertzdorf et al. 2012). Therefore, in the present study, we were interested in checking the expression of some of the inflammation-associated miRNAs in sera and blood leukocytes of sarcoidosis patients.

Interestingly, we detected significantly higher levels of miR-34a in PMBC isolated from patients with sarcoidosis when compared to healthy individuals. This miRNA has been shown to be one of the up-regulated miRNAs in melanoma cells stimulated with IFN-γ (Reinsbach et al. 2012), a cytokine characteristic for inflammatory responses and autoimmune diseases produced mostly by activated T lymphocytes (Young and Hardy 1990). Increased levels of this cytokine were detected in our analysis confirming the other observations (Asano et al. 1991; Kopinski et al. 2007). Very recently, it was shown that NF-κB signaling, involved in the stimulation of IFN-γ expression, down-regulates SIRT1 activity through miR-34a, IFN-γ, and reactive oxygen species. In turn, inhibition of SIRT1, a major regulator of energy metabolism and tissue survival, disrupts oxidative energy metabolism and stimulates the NF-κB-induced inflammatory responses (Kauppinen et al. 2013). Recently, also the role of miR-378, which tended to increase in PBMCs of sarcoidosis patients (*p* = 0.06) has been demonstrated in lung cancer (Chen et al. 2012; Skrzypek et al. 2013).

The miRNAs have been implicated both in innate and adaptive immune responses. In the present study, we were also interested in checking several other inflammation-associated miRNAs. Among them, miR-15a and miR-16 were previously shown to induce apoptosis by targeting BCL2 (Cimmino et al. 2005) and recently lower levels of these miRNAs were found in CD4^+^ T cells from relapsing–remitting multiple sclerosis patients (Lorenzi et al. 2012). The miR-155 and miR-146a were originally identified as an inflammatory response miRNAs up-regulated by NF-κB. The miR-155 targets and down-regulates the negative regulators of inflammatory response (SHIP1 and SOCS1), thus leading to increased activation of pathways dependent on Akt kinase and IFN-γ response genes, which play important roles in mediating cell survival, growth, migration, as well as antiviral responses (O’Connell et al. 2012). The miR-155 seems to be involved not only in innate, but also in adaptive immune responses and miR-155-deficient mice show marked defects in both antibody secretion and class switch recombination upon immunization (Thai et al. 2007; Vigorito et al. 2007). In contrast to miR-155, which potentiates the immune response, miR-146a is a negative regulator of the immune system. This activity of miR-146a occurs through inhibition of expression of the mRNAs encoding TRAF6 and IRAK1, two proteins that are involved in the transduction of Toll-like receptor signaling and lead to NF-κB activation (Taganov et al. 2006). As a result, miR-146a dampens the production of pro-inflammatory mediators such as IL-6 and TNF-α (Boldin et al. 2011). On the other hand, miR-150-deficient mice showed an enhanced response to immunization with T-dependent antigens. These findings are supported by additional gain-of-function studies that demonstrated a block in B-cell development dependent on c-Myb, which is a critical target of miR-150 (Xiao et al. 2007). Another miRNA, miR-192 affecting cellular proliferation through the p53 pathway, has been reported to be down-regulated in systemic lupus erythematosus, an autoimmune disease inducing inflammatory responses (Wang et al. 2011) and in asthmatic subjects upon an allergen inhalation challenge (Yamamoto et al. 2012). However, with the exception of miR-34a, in the present study, we did not find any statistically significant differences in other investigated and above-mentioned miRNAs. Further studies are ongoing to elucidate the potential roles of those miRNAs in defined cellular populations of sarcoidosis patients.

In conclusion, we provide some additional data showing that disease-related blood protein and miRNA profiles may be useful in distinguishing between sarcoidosis and healthy controls. Nevertheless, the complex interplay between miRNAs and other serum analytes involved in disease phenotypes needs to be elucidated in a larger sarcoidosis cohort, in more specified cell types, such as T regulatory lymphocytes. Global screening of miRNA transcriptome is necessary to elucidate the role of specific molecules which can be easily omitted in the analysis of selected miRNAs. Further studies are underway in which the significance of a larger panel of miRNAs, including those elucidated in the present study, will be investigated.
